# Genomic and phylogenetic characterization of severe fever with thrombocytopenia syndrome virus in companion animals in Korea, 2023–2024

**DOI:** 10.1371/journal.pntd.0014305

**Published:** 2026-06-04

**Authors:** Dong-Yeop Lee, Hong-Jae Lee, Hwi-Yeon Choi, Jason S. Park, Dong-Hun Lee

**Affiliations:** 1 Wildlife Health Laboratory, College of Veterinary Medicine, Konkuk University, Seoul, Republic of Korea; 2 Green Vet, Yongin, Republic of Korea; WRAIR, UNITED STATES OF AMERICA

## Abstract

**Background:**

Severe fever with thrombocytopenia syndrome virus (SFTSV) is a tick-borne *Phlebovirus* with high fatality rates in humans and expanding geographic distribution in East Asia. Companion animals, including dogs and cats, are increasingly recognized as susceptible hosts that may contribute to viral maintenance and spillover. However, genomic information on SFTSV strains circulating in companion animals in Korea remains limited.

**Methodology/principal findings:**

Between April 2023 and June 2024, 63 SFTSV-positive clinical specimens from dogs and cats were collected across the Republic of Korea. Whole-genome sequencing using a multiplex tiling RT-PCR and next-generation sequencing approach yielded 39 complete viral genomes. Phylogenetic analysis revealed that most viruses clustered within genotype B, including 10 viruses classified as the highly virulent B2 subtype. Five reassortant viruses (12.8%) were identified, comprising four intra-genotypic (B1/B2/B2) and one inter-genotypic (B2/C/B2) reassortants. Multiple amino acid substitutions linked to increased human case fatality, such as RdRp-T1433A (94.9%) and Gn-Q341P (97.4%), were prevalent among companion animal viruses. In addition, mutations within neutralizing antibody epitopes (Y83F, K113R, G218S, P222A, F225L, V323I, and S340N) were detected, several of which significantly reduced antibody binding affinity *in silico* (G218S, P222A, F225L, and S340N).

**Conclusions/significance:**

This study provides nationwide genomic characterization of SFTSV in companion animals in Korea. The detection of highly virulent subtypes, frequent reassortment, and epitope-altering mutations highlights the evolutionary potential of SFTSV in non-human hosts. The close genetic relationship between animal- and human-derived viruses underscores the risk of cross-species transmission. These findings emphasize the need for integrated One Health surveillance systems linking veterinary and human health sectors to enable early detection, risk assessment, and mitigation of emerging SFTSV threats.

## Author summary

Severe fever with thrombocytopenia syndrome virus (SFTSV) is a tick-borne virus that can cause severe disease and death in humans across East Asia. Dogs and cats are also susceptible, but little is known about the viruses circulating in these animals. We analyzed samples from 63 infected dogs and cats across Korea between 2023 and 2024 and obtained 39 complete viral genomes. Most belonged to genotype B, including ten viruses of the highly virulent B2 subtype that has also been linked to fatal human infections. We detected frequent genetic reassortment events (5/39, 12.8%), including both within-genotype and cross-genotype reassortants. Several common mutations were identified in proteins involved in viral replication and immune recognition, such as RdRp-T1433A and Gn-Q341P, which have been associated with higher human fatality, as well as changes in antibody target sites that may reduce immune protection. Importantly, the animal viruses were genetically close to those found in human patients, suggesting that cross-species transmission is possible. These results highlight the role of companion animals in the ecology of SFTSV and the importance of One Health surveillance linking veterinary and human medicine.

## Introduction

Severe Fever with Thrombocytopenia Syndrome Virus (SFTSV), a member of the species *Bandavirus dabieense* within the genus *Bandavirus* (family *Phenuiviridae*, order *Bunyavirales*), is a significant emerging tick-borne pathogen [1]. Initially identified in China in 2009, SFTSV has subsequently been documented across multiple countries in East Asia, including the Republic of Korea and Japan, with increasing annual incidence and a human case fatality rate [2–6]. More recently, cases have also been reported in additional regions, including Vietnam, Thailand, Myanmar, and Pakistan, suggesting an expanding geographic distribution of SFTSV [5,7–9]. The virus is primarily transmitted through *Haemaphysalis* spp. ticks, although human-to-human transmission via direct contact with infected blood has been documented [10,11]. Clinical presentation of SFTSV infection is characterized by fever, thrombocytopenia, leukopenia, and multi-organ dysfunction, with case fatality rates varying from 16% to 40% [12]. The absence of specific antivirals and vaccines highlights the importance of understanding the molecular and genetic characteristics of the virus, which are critical not only for the development of targeted intervention strategies but also for improving genomic surveillance and assessing potential transmission risks [13,14].

SFTSV possesses a negative-sense, single-stranded RNA genome consisting of three segments. The L segment encodes the RNA-dependent RNA polymerase (RdRp), which mediates viral replication. The M segment encodes glycoproteins (Gn and Gc) that facilitate host cell entry and interact with the host immune system. The S segment encodes both the nucleocapsid protein (NP) and the non-structural protein (NS), which plays a role in immune evasion [2,15]. Genomic variation across these segments has been demonstrated to influence viral pathogenicity, with distinct genetic lineages identified through phylogenetic analyses, suggesting potential variations in virulence and epidemiological patterns [16,17].

SFTSV demonstrates remarkable host range versatility, with evidence of infection documented in both domestic and wild animal populations [18]. While arthropod vectors, primarily ticks, constitute the predominant transmission route to humans and non-human animals, transmission via contact with infectious blood or body fluids has also been reported [19,20]. Inter- and intra-species transmission events (including human-to-human, animal-to-human, and animal-to-animal) have been documented during SFTSV outbreaks [21–23]. Notably, companion animals such as dogs (*Canis lupus familiaris*) and cats (*Felis catus*) have been shown to develop clinical signs of SFTS similar to those observed in humans. Moreover, the detection of high viral loads in their body fluids raises concern about their potential role as amplifying hosts, facilitating zoonotic spillover and complicating containment efforts [24,25]. The expanding host tropism and transmission dynamics of SFTSV present significant challenges for public and veterinary health systems.

From April 2023 to June 2024, clinical specimens from suspected SFTSV cases were collected from veterinary medical hospitals throughout the Republic of Korea. Of these, 63 were confirmed SFTSV-positive through quantitative polymerase chain reaction (qPCR) analysis conducted at Green Vet (Yongin, Gyeonggi-do, Republic of Korea). This study aimed to conduct a comprehensive genomic characterization of SFTSV from companion animals (dogs and cats), with a focus on genetic diversity assessment, phylogenetic reconstruction, and identification of potential mutations. Through the application of next-generation sequencing (NGS) and bioinformatic analyses, we characterized the genomic features and phylogenetic relationships of SFTSV in companion animals and provide insights into its potential implications for public health and surveillance.

## Materials and methods

### Sample collection and viral RNA preparation

Clinical specimens were collected from companion dogs and cats suspected of SFTSV infection, exhibiting clinical signs such as fever, thrombocytopenia, anorexia, and lethargy, between April 2023 and June 2024. The canine subjects represented 20 breeds (Bichon Frise, Cairn Terrier, Cardigan Welsh Corgi, Cavalier King Charles Spaniel, Cocker Spaniel, Coton de Tulear, Finnish Spitz, Golden Retriever, Japanese Spitz, Labrador Retriever, Maltese, Maltipoo, Miniature Schnauzer, Mixed, Pomeranian, Poodle, Samoyed, Shiba Inu, Shih-Tzu, and Yorkshire Terrier), while the feline case was a Korean Shorthair. Samples were obtained from nine provinces (Gyeonggi, Gangwon, North and South Chungcheong, North and South Gyeongsang, North and South Jeolla, and Jeju) and eight metropolitan cities (Seoul, Busan, Daegu, Daejeon, Gwangju, Incheon, Sejong, and Ulsan) in the Republic of Korea. Detailed information regarding the total clinical specimens and SFTSV-positive samples is presented in S1 Table.

Whole blood samples from each suspected animal were collected in sterile EDTA K3 blood collection tubes (AB MEDICAL Co., Ltd., Republic of Korea) and transported to Green Vet (Yongin, Gyeonggi-do, Republic of Korea) under constant 4 °C conditions.

A 200 μL aliquot of each sample was used for viral nucleic acid extraction using the MagNA Pure 96 DNA and Viral NA Small Volume Kit (Roche, Switzerland) and the MagNA Pure 96 fully automated nucleic acid extraction system (Roche, Switzerland), following the manufacturer’s instructions. Purified nucleic acids were eluted in 50 μL of elution buffer.

To identify SFTSV-positive specimens, 5 μL of each eluted nucleic acid was subjected to multiplex qPCR using the GCani Canine/Feline Anemia qPCR Detection Kit and the GCani SFTSV qPCR Detection Kit (Genes Laboratories, Republic of Korea) on a CFX96 Real-Time PCR Detection System (BIO-RAD, CA, USA), following the manufacturer’s instructions. The spectrum of pathogens detectable by each kit is presented in S2 Table.

Briefly, the PCR mixture for the GCani Canine/Feline Anemia qPCR Detection Kit was prepared by combining 10 μL of the Anemia master mixture, 5 μL of the Anemia PP mixture, and 5 μL of either the clinical sample or positive control template. 5 μL of nuclease-free water was used for the negative control template. Similarly, the PCR mixture for the GCani SFTSV qPCR Detection Kit was prepared by mixing 10 μL of the SFTSV master mixture, 5 μL of the SFTSV PP mixture, and 5 μL of the clinical sample or positive control template. 5 μL of nuclease-free water was used for the negative control template. The thermal cycling conditions for each assay were as follows: GCani Canine/Feline Anemia qPCR Detection Kit: initial denaturation (95 °C, 5 min), followed by 40 cycles of denaturation at 95 °C for 15 sec and annealing/extension at 58 °C for 1 min. GCani SFTSV qPCR Detection Kit: reverse transcription (50 °C, 20 min), initial denaturation (95 °C, 5 min), followed by 40 cycles of denaturation at 95 °C for 15 sec and annealing/extension at 58 °C for 1 min. Fluorescence signals were analyzed using CFX Manager Dx Software v3.1. Samples with a cycle threshold (Ct) value below 40 were considered positive for the corresponding pathogens.

Clinical specimens and purified viral nucleic acids confirmed as SFTSV-positive were stored at –80 °C in the Green Vet Clinical Biobank (GVCB) after the exclusion of personal information of animal patients and clients, for further analysis and whole-genome sequencing.

### Primer design and tiling amplicon PCR for whole-genome sequencing

A multiplex tiling reverse transcription polymerase chain reaction (RT-PCR) methodology was developed for whole-genome sequencing of SFTSV from clinical specimens. Primers were designed using PrimalScheme [26] based on multiple sequence alignment of all available SFTSV genomes originating from the Republic of Korea in the National Center for Biotechnology Information (NCBI) Virus database. The primer panel included 14 pairs for the L segment, 8 pairs for the M segment, and 5 pairs for the S segment. The primer sequences used in this study are provided in S3 Table.

RT-PCR was performed on 63 SFTSV-positive clinical samples using the OneStep RT-PCR Kit (Qiagen, Hilden, Germany) following the manufacturer’s instructions. The PCR mixture consisted of 10 μL 5x QIAGEN OneStep RT-PCR buffer, 2 μL dNTP mix (10 mM), 29.5 μL nuclease-free water, 2 μL QIAGEN OneStep RT-PCR Enzyme Mix, 4 μL template RNA, and 2.5 μL primer mix, with a final concentration of 0.1 μM for each primer, except for the M-8-L/R (0.4 μM) to ensure full genome coverage. The PCR program consisted of reverse transcription (50 °C, 30 min), initial denaturation (95 °C, 15 min), 40 cycles of denaturation/annealing/extension (94 °C/1 min, 58 °C/1 min, and 72 °C/1 min, respectively), and final extension (72 °C, 10 min). Amplicons (458–709 bp) were confirmed on 1% agarose gels, pooled, and sequenced on the Illumina MiniSeq platform (Illumina Inc., San Diego, CA, USA). Sequencing yielded a minimum of 250,000 paired-end reads (150 bp) per sample with mean coverage exceeding 1,700× across the viral genome.

### Genome assembly and phylogenetic analysis

Sequence reads were processed using BBDuk (v38.84) with a quality threshold Q30 and a minimum length of 50 bp. Viral genomes were assembled both *de novo* using Geneious assembler (v2024.0.4, Biomatters Ltd., Auckland, New Zealand) and by reference-mapping to CB1 strain (GenBank accession No.: L segment - KY789433, M segment - KY789436, and S segment - KY789439) using Minimap (v2.24) with default parameters [27].

Genetic analyses utilized complete SFTSV genome sequences retrieved from the BV-BRC database (accessed on January 27, 2025). Strains with all three full-length segments were included in the phylogenetic analysis alongside sequences obtained in this study. Multiple sequence alignment was conducted using MAFFT (v7.490) [28] and sequences with > 99.7% nucleotide identity were eliminated using ElimDupes (https://www.hiv.lanl.gov/content/sequence/elimdupesv2/elimdupes.html). Maximum-likelihood (ML) phylogenetic analyses for L, M, and S segments were constructed using IQ-TREE 2 with automatic model selection [29]. Node support was assessed using 1,000 ultrafast bootstrap replicates. Phylogenetic trees were visualized in Interactive Tree of Life (iTOL, v7.1.1) [30]. SFTSV genotypes of each segment were classified according to the genotyping system described in previous studies [14,17].

### Recombination and reassortment analysis

Maximum-likelihood phylogenetic trees were reconstructed for the complete L, M, and S segments using strains with all three full-length segments to generate the tanglegram visualization. Phylogenetic inference was performed using FastTree (v2.1.11) under the GTR + Γ substitution model [31]. The resulting segment trees were visualized as a tanglegram using Baltic (v0.3.0), with custom Python scripts adapted from Kim et al. [32].

Potential recombination events were analyzed using the Recombination Detection Program (RDP, v4.101) [33]. Seven detection methods (3Seq, Bootscan, Chimaera, GENECONV, MaxChi, RDP, and SiScan) were utilized. A recombination event was considered authentic if it was detected by at least five detection methods with a significance threshold of p < 0.05.

### Amino acid mutation analysis

Amino acid mutations previously associated with increased SFTS case fatality in humans were used as reference positions for comparative screening. A list of 25 significant amino acid substitutions identified from previous study was extracted, covering five NS, nine Gn, four Gc, and seven RdRp positions [34]. Whole-genome sequences obtained in this study were translated to amino acid sequences using Geneious Prime (v2025.1.2) and aligned using MAFFT (v7.490) [28]. The presence or absence of these mutations in each of the 39 SFTS viruses was manually curated.

### Selection pressure analysis

Selection pressure analysis was conducted for each coding region of the SFTSV genome using the Datamonkey platform [35]. The nonsynonymous (dN) versus synonymous (dS) nucleotide ratio (dN/dS) within the sequence was calculated using the single-likelihood ancestor counting (SLAC) implemented in Datamonkey. Codons were categorized as being under neutral selection (dN/dS = 1), positive/diversifying selection (dN/dS > 1), or negative/purifying selection (dN/dS < 1). To further identify codons under positive selection, additional analyses were performed using the Fast Unconstrained Bayesian AppRoximation (FUBAR), Mixed Effects Model of Evolution (MEME), and Fixed-Effects Likelihood (FEL) methods. For SLAC, FEL, and MEME, only codon sites with statistically significant P-values (< 0.1) were considered positively selected, whereas for FUBAR, codons with a posterior probability > 0.9 were interpreted as being under positive selection pressure. Codon sites identified by at least two of the four methods were considered to be under significant positive selection.

### Epitope sequence variation analysis

Based on a recent study, three novel epitopes within the Gn-head domain targeted by Gn-specific monoclonal antibodies were identified, and key mutations within these epitopes were shown to reduce antibody neutralization activity [36]. To further investigate sequence variability in these regions, we aligned the amino acid sequences of the Gn-head domain from viruses analyzed in this study with the reference strain WCH/97/HN/China/2011 (GenBank accession No.: JQ341189) using MAFFT (v7.490) [28]. Amino acid differences within the identified epitope regions were examined, and mutations potentially associated with reduced antibody binding were identified.

### Molecular docking analysis

To assess the effect of mutations within the Gn-head domain epitope regions on antibody binding affinity, molecular docking analysis was performed. First, amino acid substitutions identified in the epitope sequence variation analysis were introduced into the Gn-head domain of the reference strain WCH/97/HN/China/2011 to generate mutated sequences, which were subsequently subjected to 3D structure prediction.

Structural models were generated using I-TASSER [37], SWISS-MODEL [38], ColabFold [39], and AlphaFold3 [40], and their quality was compared using MolProbity [41]. Among these, AlphaFold3 produced the most reliable structural model and was therefore selected for further analysis. The heavy and light chain sequences of the monoclonal antibodies reported in the previous study were retrieved from the RCSB PDB database, and their 3D structures were predicted using AlphaFold3. The structures were visualized using PyMOL (v3.1.0) [42].

Molecular docking analysis was performed using HADDOCK (v2.5) [43]. Each monoclonal antibody was docked to the corresponding Gn-head domain following the HADDOCK 2.4 antibody-antigen docking tutorial [44]. For antibody structures, complementarity-determining region (CDR) residues were identified using AbodyBuilder2 and defined as active residues [45]. Epitope residues within the Gn-head domain, previously reported in the literature [36], were designated as passive residues. Docking metrics were extracted from the complete run file in each HADDOCK docking analysis. Interface residues were defined as residues within 5 Å of any atom of the docking partner in the final complex structures [46].

### Statistics

Nonparametric statistical methods were employed to assess differences in antibody binding affinity. Wilcoxon rank-sum tests were applied to compare binding affinities across different mutations. All statistical analyses were conducted using R (v4.5.1) [47], and visualizations were produced with the ggplot2 (v4.0.2) [48] and ggsignif (v0.6.4) [49] packages.

## Results

### Genome sequencing

**Fig 1:**
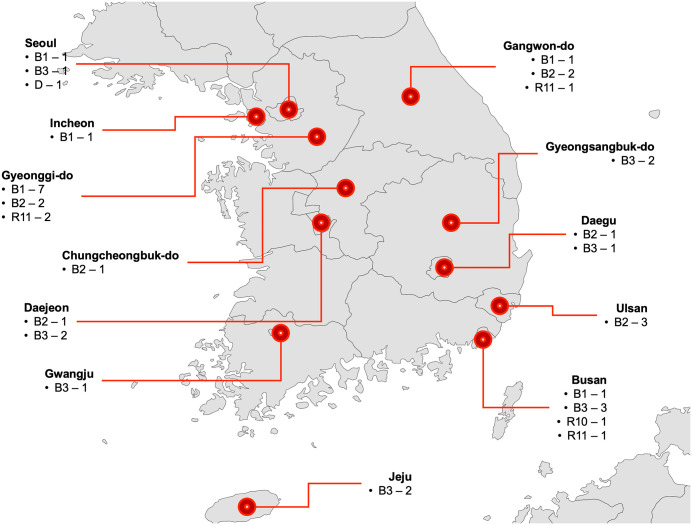
Geographic distribution of SFTSV genotypes identified from companion animals in the Republic of Korea. Sampling sites and associated genotypes are shown across administrative regions. The number of viruses per genotype is indicated for each location. Made with Natural Earth (https://www.naturalearthdata.com/downloads/10m-cultural-vectors/).

We successfully obtained 39 (62%) complete genome sequences from 63 SFTSV-positive clinical samples of companion animals using multiplex tiling RT-PCR and NGS (Fig 1) [26]. Based on the performance of our tiling RT-PCR protocol, samples with Ct values as high as 35.11 could be sequenced; however, reliable whole-genome assembly was generally achieved for samples with Ct values below 33 in our dataset, suggesting a practical threshold for successful genome recovery from clinical material (S4 Table). The whole-genome sequences of the SFTS viruses obtained in this study were deposited into the NCBI database (GenBank accession No.: PV704039-PV704155).

The 39 complete genome sequences shared nucleotide identities of ≥ 95.60% in the L, ≥ 93.33% in the M, and ≥ 94.68% in the S segment (S1 Fig). Compared to the CB1 reference strain (GenBank accession No.: KY789433, KY789436, and KY789439), the nucleotide sequences showed ≥ 95.73% identity in the L, ≥ 94.35% in the M, and ≥ 94.80% in the S segment.

### BLAST search and phylogenetic analysis

BLAST analysis revealed that the consensus sequence of the L segment shared the highest nucleotide identity (≥ 96.29%) with the strain 15KS75 (GenBank accession No.: MG921169), a human-derived serum sample collected in the Republic of Korea in 2015. The M segment consensus sequence was most similar to the strain JN13 (GenBank accession No.: OQ644293) (≥ 94.13%), a human-derived serum sample in the Republic of Korea in 2019. The S segment consensus sequence showed the highest similarity to the strain 15KS23 (GenBank accession No.: MG737240) (≥ 96.24%), a human-derived serum sample in the Republic of Korea in 2015.

**Fig 2:**
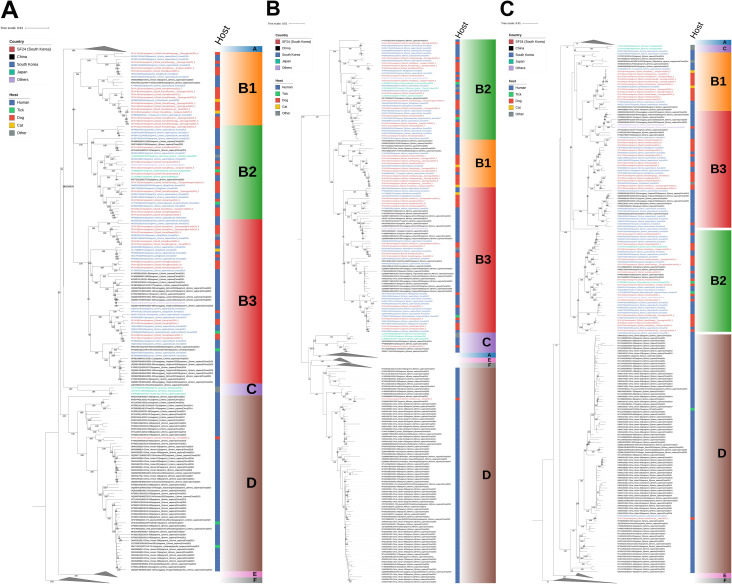
Maximum-likelihood phylogenetic trees of the L(A), M (B), and S (C) segments of SFTS viruses from this study. The scale bar represents the number of substitutions per site. Ultrafast bootstrap (UFBoot) values (≥ 95) are shown at the corresponding nodes. Viruses from this study are highlighted in red. Taxon label colors indicate the country of origin, while the color strip on the right denotes host species. Genotypes are indicated by colored ranges. Genotypes not associated with the viruses from this study (A, E, and F) were collapsed. All trees are midpoint-rooted‌‌.

Maximum-likelihood phylogenetic analyses of the L, M, and S segments revealed that the majority of SFTSV sequences obtained in this study clustered within genotype B (B1 = 11, B2 = 10, B3 = 12) and one virus (SF24–72) grouped with genotype D (n = 1) (Figs 2, S2) [17,50].

### Recombination and reassortment analysis

**Fig 3:**
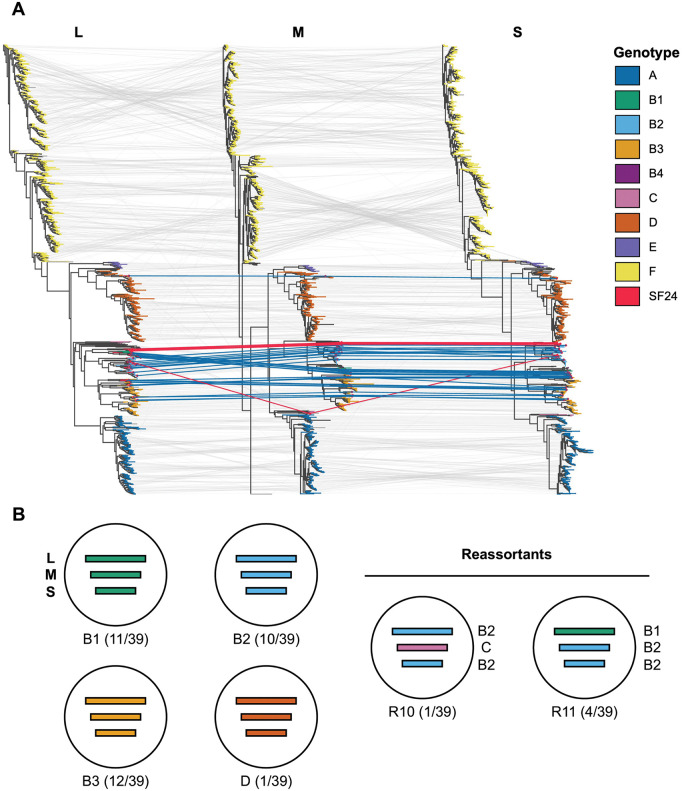
Tanglegram and genotype constellations of SFTS viruses identified in this study. **(A)** Tanglegram illustrating the phylogenetic relationships of the L, M, and S genome segments. Terminal branches are colored according to genotype. Lines connecting identical strains across the segment trees indicate the correspondence of strains among the three segment phylogenies. Viruses from this study are indicated by blue lines, whereas previously reported strains are shown in gray; reassortant viruses identified in this study are highlighted in red. **(B)** Genotype constellations and reassortment patterns identified in this study. Each genotype constellation is represented by three horizontal bars corresponding to the L, M, and S genome segments. Bar colors indicate the genotype assigned to each segment. The numbers in parentheses denote the number of viruses belonging to each constellation among the 39 genomes analyzed.

Phylogenetic comparison of the L, M, and S segments revealed that several viruses exhibited discordant clustering across the segment trees (Figs 2–3), identifying 5/39 (12.8%) reassortant viruses (S4 Table). Among them, four viruses (SF24–2, 6, 57, and 74) showed intra-genotype reassortment within genotype B, corresponding to the previously reported R11 genotype constellation (B1/B2/B2) [14,17]. Notably, SF24–19 virus demonstrated inter-genotypic reassortment: the L and S segments clustered within genotype B2, whereas the M segment clustered within genotype C (B2/C/B2, corresponding to R10), indicating a reassortment event between genetically distinct viral lineages.

No recombination events were identified in any of the genomic segments of SFTSV in our clinical samples.

### Virulence-associated amino acid mutations

Among the 39 complete genome sequences obtained from this study, several amino acid substitutions previously associated with increased fatality in human cases were identified (Table 1) [34]. In the S segment, NS-I223V (SF24–72) and D239E (SF24–14) mutations were each detected in 1/39 viruses (2.6%). In the M segment, Gn-Q341P and Gn-I491M were present in 38/39 (97.4%) and 29/39 (74.4%) viruses, respectively, while Gn-R525G was observed in 3/39 (7.7%) viruses (SF24–7, SF24–9, and SF24–72). Notably, SF24–72 virus showed the highest accumulation of mutations within the Gn protein, harboring five substitutions (D37G, T273A, Q341P, I491M, and R525G). In the L segment, RdRp-T1433A and I1447V were identified in 37/39 (94.9%) and 15/39 (38.5%) viruses, respectively, whereas RdRp-R1684K was detected in 2/39 viruses (5.1%; SF24–20 and SF24–85).

**Table 1:** Virulence-associated amino acid mutations identified in SFTS viruses from this study.

Protein	Genotype	NS**		Gn					RdRp			M/T
OR		2.072	2.626	2.139	1.993	2.077	1.84	1.944	2.847	1.932	2.986	
Mutation*		I223V	D239E	D37G	T273A	Q341P	I491M	R525G	T1433A	I1447V	R1684K	
**SF24–1**	B2	I	D	N	T	**P**	**M**	R	**A**	I	R	3/25
**SF24–2**	Reassortant (R11)	I	D	N	T	**P**	V	R	**A**	**V**	R	3/25
**SF24–3**	B1	I	D	N	T	**P**	I	R	**A**	**V**	R	3/25
**SF24–4**	B1	I	D	N	T	**P**	**M**	R	**A**	**V**	R	4/25
**SF24–5**	B1	I	D	N	T	**P**	I	R	**A**	**V**	R	3/25
**SF24–6**	Reassortant (R11)	I	D	N	T	**P**	V	R	**A**	**V**	R	3/25
**SF24–7**	B3	I	D	N	T	**P**	**M**	**G**	**A**	I	R	4/25
**SF24–8**	B2	I	D	N	T	**P**	**M**	R	**A**	I	R	3/25
**SF24–9**	B3	I	D	N	T	**P**	**M**	**G**	**A**	I	R	4/25
**SF24–10**	B1	I	D	N	T	**P**	**M**	R	**A**	**V**	R	4/25
**SF24–11**	B2	I	D	N	T	**P**	**M**	R	**A**	I	R	3/25
**SF24–12**	B1	I	D	N	T	**P**	**M**	R	**A**	**V**	R	4/25
**SF24–13**	B1	I	D	N	T	**P**	I	R	**A**	**V**	R	3/25
**SF24–14**	B2	I	**E**	N	T	S	**M**	R	**A**	I	R	3/25
**SF24–15**	B1	I	D	N	T	**P**	**M**	R	**A**	**V**	R	4/25
**SF24–16**	B2	I	D	S	T	**P**	**M**	R	**A**	I	R	3/25
**SF24–17**	B2	I	D	N	T	**P**	**M**	R	**A**	I	R	3/25
**SF24–18**	B2	I	N	N	T	**P**	**M**	R	**A**	I	R	3/25
**SF24–19**	Reassortant (R10)	I	D	D	T	**P**	**M**	R	**A**	I	R	3/25
**SF24–20**	B3	I	D	N	T	**P**	**M**	R	**A**	I	**K**	4/25
**SF24–42**	B3	I	D	N	T	**P**	**M**	R	**A**	I	R	3/25
**SF24–53**	B1	I	D	N	T	**P**	I	R	**A**	**V**	R	3/25
**SF24–55**	B2	I	D	N	T	**P**	**M**	R	**A**	I	R	3/25
**SF24–56**	B1	I	D	N	T	**P**	**M**	R	**A**	**V**	R	4/25
**SF24–57**	Reassortant (R11)	I	D	N	T	**P**	V	R	**A**	**V**	R	3/25
**SF24–59**	B3	I	D	N	T	**P**	**M**	R	**A**	I	R	3/25
**SF24–60**	B3	I	D	N	T	**P**	**M**	R	**A**	I	R	3/25
**SF24–62**	B1	I	D	N	T	**P**	I	R	**A**	**V**	R	3/25
**SF24–64**	B1	I	D	N	T	**P**	I	R	**A**	**V**	R	3/25
**SF24–66**	B2	I	D	N	T	**P**	**M**	R	**A**	I	R	3/25
**SF24–67**	B3	I	D	N	T	**P**	**M**	R	**A**	I	R	3/25
**SF24–68**	B3	I	G	N	T	**P**	**M**	R	**A**	I	R	3/25
**SF24–69**	B3	I	D	N	T	**P**	**M**	R	**A**	I	R	3/25
**SF24–70**	B3	I	D	N	T	**P**	**M**	R	**A**	I	R	3/25
**SF24–72**	D	**V**	D	**G**	**A**	**P**	**M**	**G**	T	I	R	6/25
**SF24–73**	B2	I	D	N	T	**P**	**M**	R	T	I	R	2/25
**SF24–74**	Reassortant (R11)	I	D	N	T	**P**	V	R	**A**	**V**	R	3/25
**SF24–78**	B3	I	D	N	T	**P**	**M**	R	**A**	I	R	3/25
**SF24–85**	B3	I	D	N	T	**P**	**M**	R	**A**	I	**K**	4/25

* Mutations previously linked to enhanced virulence are indicated in bold.

** NS, non-structural protein; Gn/Gc, glycoproteins; RdRp, RNA-dependent RNA polymerase; OR, odds ratio; M/T, matched/total.

### Selection pressure analysis

The dN/dS ratios were 0.0533 for RdRp, 0.0983 for the glycoprotein, 0.035 for NP, and 0.116 for NS (Table 2). Significant positive selection was detected at six codon sites in RdRp (2, 691, 719, 1061, 1116, and 1353), eight sites in the glycoprotein (8, 9, 13, 37, 170, 298, 323, and 501), and five sites in NS (145, 249, 272, 281, and 289). No codon sites under significant positive selection were detected in NP.

**Table 2:** Molecular selection pressure of the SFTSV genomes.

Gene segment	Coding region	Method	Number of positive selection codon sites (location of sites)	Number of negative selection sites (%)	dN/dS
L	RdRp*	SLAC	5 (2, 691, 719, 1061, 1353)	1257 (60.3)	0.0533
		FEL	7 (2, 691, 713, 719, 1061, 1116, 1353)	1548 (74.3)	
		FUBAR	7 (2, 479, 691, 719, 1061, 1116, 1353)	1843 (88.4)	
		MEME	26 (2, 4, 7, 8, 223, 520, 652, 669, 670, 719, 1061, 1097, 1116, 1202, 1353, 1355, 1375, 1404, 1405, 1608, 1675, 1684, 1875, 1896, 1960, 2013)	–	
M	Glycoprotein	SLAC	7 (8, 13, 37, 170, 298, 323, 501)	658 (61.3)	0.0983
		FEL	12 (8, 9, 13, 170, 298, 323, 404, 501, 530, 803, 984, 1063)	763 (71.1)	
		FUBAR	8 (8, 13, 37, 170, 298, 323, 501, 1011)	871 (81.2)	
		MEME	17 (8, 9, 13, 154, 170, 187, 274, 278, 298, 323, 501, 524, 630, 743, 956, 1018, 1053)	–	
S	NP	SLAC	0	130 (53.1)	0.035
		FEL	0	179 (73.1)	
		FUBAR	0	190 (77.6)	
		MEME	1 (212)	–	
	NS	SLAC	2 (145, 289)	172 (58.7)	0.116
		FEL	6 (145, 238, 249, 272, 281, 289)	203 (69.3)	
		FUBAR	4 (145, 249, 281, 289)	220 (75.1)	
		MEME	11 (79, 112, 122, 123, 145, 150, 151, 249, 272, 281, 289)	–	

* RdRp, RNA-dependent RNA polymerase; NP, nucleocapsid protein; NS, non-structural protein; dN/dS, nonsynonymous versus synonymous nucleotide ratio.

### Epitope sequence variation analysis

Comparison of the epitope amino acid sequences revealed only a few variations between the Gn-head domain of the reference strain WCH/97/HN/China/2011 and the viruses analyzed in this study (Table 3). A K113R substitution was identified within the epitope region targeted by the S2A5 antibody. Mutations within the N1D10 antibody epitope included Y83F, G218S, P222A, F225L, and S340N. A single substitution, V323I, was observed in the B1G11 antibody target site.

**Table 3:** Summary of the amino acid substitutions at monoclonal antibody-targeted epitope residues in the SFTSV Gn-head domain.

Antibody	Genotype	N1D10	S2A5	N1D10	B1G11	N1D10
Position (Length)		83 (1)	111-114 (4)	218-226 (9)	321-323 (3)	339-340 (2)
Reference *		Y	KAKG	GESLPQPFD	MRV	VS
SF24–1	B2	F	KAKG	SESLPQPFD	MRV	VN
SF24–2	Reassortant (R11)	F	KAKG	SESLPQPFD	MRV	VN
SF24–3	B1	Y	KAKG	SESLPQPFD	MRV	VN
SF24–4	B1	Y	KAKG	SESLPQPFD	MRV	VN
SF24–5	B1	Y	KAKG	SESLPQPFD	MRV	VN
SF24–6	Reassortant (R11)	F	KAKG	SESLPQPFD	MRV	VN
SF24–7	B3	Y	KAKG	SESLAQPFD	MRV	VN
SF24–8	B2	F	KAKG	SESLPQPFD	MRV	VN
SF24–9	B3	Y	KAKG	SESLAQPFD	MRV	VN
SF24–10	B1	Y	KAKG	SESLPQPFD	MRV	VN
SF24–11	B2	F	KAKG	SESLPQPFD	MRV	VN
SF24–12	B1	Y	KAKG	SESLPQPFD	MRV	VN
SF24–13	B1	Y	KAKG	SESLPQPFD	MRV	VN
SF24–14	B2	F	KAKG	SESLPQPFD	MRV	VN
SF24–15	B1	Y	KAKG	SESLPQPFD	MRV	VN
SF24–16	B2	F	KARG	SESLPQPFD	MRV	VN
SF24–17	B2	F	KAKG	SESLPQPFD	MRV	VN
SF24–18	B2	F	KAKG	SESLPQPFD	MRV	VN
SF24–19	Reassortant (R10)	Y	KAKG	GESLPQPFD	MRI	VN
SF24–20	B3	Y	KAKG	SESLPQPLD	MRV	VN
SF24–42	B3	Y	KAKG	SESLPQPLD	MRV	VN
SF24–53	B1	Y	KAKG	SESLPQPFD	MRV	VN
SF24–55	B2	F	KAKG	SESLPQPFD	MRV	VN
SF24–56	B1	Y	KAKG	SESLPQPFD	MRV	VN
SF24–57	Reassortant (R11)	F	KAKG	SESLPQPFD	MRV	VN
SF24–59	B3	Y	KAKG	SESLPQPFD	MRV	VN
SF24–60	B3	Y	KAKG	SESLPQPFD	MRV	VN
SF24–62	B1	Y	KAKG	SESLPQPFD	MRV	VN
SF24–64	B1	Y	KAKG	SESLPQPFD	MRV	VN
SF24–66	B2	F	KAKG	SESLPQPLD	MRV	VN
SF24–67	B3	Y	KAKG	SESLAQPFD	MRV	VN
SF24–68	B3	Y	KAKG	SESLPQPFD	MRV	VN
SF24–69	B3	Y	KAKG	SESLPQPFD	MRV	VN
SF24–70	B3	Y	KAKG	SESLPQPFD	MRV	VN
SF24–72	D	Y	KAKG	GESLPQPFD	MRV	VN
SF24–73	B2	F	KAKG	SESLPQPFD	MRV	VN
SF24–74	Reassortant (R11)	F	KAKG	SESLPQPFD	MRV	VN
SF24–78	B3	Y	KAKG	SESLPQPFD	MRV	VN
SF24–85	B3	Y	KAKG	SESLPQPFD	MRV	VN
Critical residue **		Y83	K111			

* WCH/97/HN/China/2011 (GenBank accession No.: JQ341189)

** Positions showing > 70% reduction in binding upon alanine substitution compared with wild-type Gn.

Three-dimensional structure prediction of the Gn-head protein indicated that the mutations identified in our viruses did not induce significant conformational changes, as indicated by root mean square deviation (RMSD) values ranging from 0.169 to 0.247 Å.

Molecular docking analysis identified interface residues (≤5 Å) at the antibody binding sites for each monoclonal antibody (S3 Fig, S5 Table). Key amino acid mutations detected through epitope sequence variation analysis were in close proximity to the antibody binding interfaces, and all were included among the identified interface residues, namely TYR83, LYS113, GLY218, PRO222, PHE225, VAL323, and SER340.

**Fig 4:**
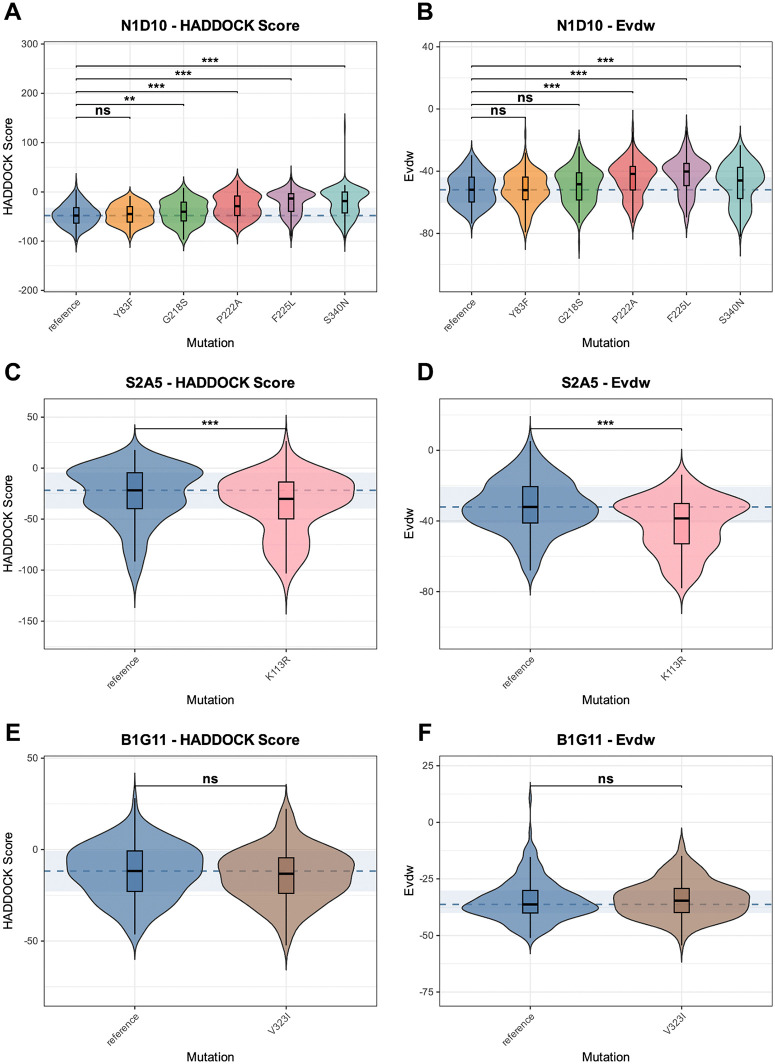
Effects of Gn-head domain mutations on antibody binding affinity predicted by molecular docking analysis. Violin plots show the distributions of HADDOCK scores and van der Waals energies (Evdw) for mutations within the epitope regions of N1D10 **(A, B)**, S2A5 **(C, D)**, and B1G11 **(E, F)**. The dashed horizontal line indicates the median of the reference group, and the shaded region represents the interquartile range (IQR) of the reference. Internal box plots show the median, Q1-Q3, and 1.5 × IQR whiskers. Lower HADDOCK scores and more negative Evdw values indicate stronger predicted binding affinity. Statistical comparisons between the reference and each variant were performed using the Wilcoxon rank-sum test with Benjamini–Hochberg false discovery rate (FDR) correction. Significance levels are based on FDR-adjusted p–values: *p < 0.05; **p < 0.01; ***p < 0.001; ns (not significant), p ≥ 0.05.

Several polymorphisms within the Gn-head domain epitope regions significantly affected antibody binding affinity *in silico* (Fig 4). For the N1D10 epitope, the P222A, F225L, and S340N mutations resulted in significant increases in both the HADDOCK score and van der Waals energy (Evdw), indicating reduced binding affinity. The G218S mutation also caused a significant increase in the HADDOCK score, suggesting reduced binding affinity. In the S2A5 region, the K113R mutation produced a significant decrease in both the HADDOCK score and Evdw, indicating increased binding affinity. In contrast, the V323I substitution within the B1G11 epitope showed no significant difference in binding affinity compared with the reference.

## Discussion

SFTS, caused by *Bandavirus dabieense*, continues to pose a serious public health threat in East Asia due to its expanding geographic range, high case fatality rate, and evidence of both vector-borne and non-vector transmission routes [2–6,22,51]. The increasing incidence of SFTS has been attributed to multiple factors, including changes in transmission dynamics involving reservoirs and vectors (e.g., ticks and animals), shifts in wildlife ecology or environmental conditions driven by climate change, and improvements in diagnostic detection methods for SFTS infection [52,53]. Given that companion animals can serve as a reservoir for SFTS infection due to their close interaction with humans [54,55], it is important to monitor SFTSV prevalence and analyze the epidemiological characteristics of this virus in companion animals. During the period from April 2023 to June 2024 in the Republic of Korea, both the total number of SFTSV-positive clinical cases from companion animals and the positivity rate increased. This seasonal increase is consistent with the epidemiological pattern observed in human and other animal SFTS cases, which typically peak during periods of high tick activity [56–58]. The positivity rate, derived from clinical data provided by Green Vet, was 1.21% (20 of 1,659 suspected cases) in 2023 and 1.96% (43 of 2,197 suspected cases) in 2024. Despite increasing reports of SFTSV infection in both humans and animals, the genetic diversity and molecular characteristics of the virus in companion animals remain relatively underexplored, particularly in the Republic of Korea.

In this study, we applied a tiling amplicon-based NGS approach to investigate SFTSV infections in dogs and cats presenting with clinical signs [26]. In total, we successfully recovered complete viral genomes from 62% (39/63) of the qPCR-positive clinical samples. This recovery rate was likely influenced by the amount and quality of viral RNA in clinical specimens. Our results demonstrate that whole-genome sequences can be recovered from clinical samples with Ct values as high as 35.11, highlighting the applicability of this protocol in field surveillance of SFTSV and its potential utility for genomic risk assessment.

Phylogenetic analysis revealed that most sequenced viruses clustered within genotype B, consistent with the predominant genotypes circulating in the Republic of Korea [17,26,50]. Notably, 10 of the 39 viruses were identified as genotype B2, which has previously been associated with the highest case fatality rates among SFTSV strains [14]. The identification of these genotypes in companion animals suggests that dogs and cats may serve as potential reservoirs of highly pathogenic strains, posing a risk for zoonotic transmission. Moreover, most viruses showed high sequence similarity and close phylogenetic clustering with human-derived strains from the Republic of Korea, indicating regional endemic pattern and the potential for interspecies transmission. While animal-to-human transmission of SFTSV has been reported [54,55], these observations raise the possibility of bidirectional transmission between humans and companion animals, as observed in other zoonotic viruses [59–61].

The segmented nature of the SFTSV genome facilitates frequent reassortment events, contributing to its genetic diversity [17,50,62–65]. In our dataset, we identified 5/39 (12.8%) reassortant viruses, including four intra-genotypic reassortants within genotype B —R11 (B1/B2/B2)— and one inter-genotypic reassortant —R10 (B2/C/B2) [14,17]. The R11 reassortant type has been previously linked to higher case fatality rates in human cases reported between 2012 and 2017, resulting in a case fatality rate of 50% (2 out of 4 cases), and 100% lethality in aged ferret models, suggesting increased virulence [17]. Importantly, this reassortant type was the most frequently detected in the Republic of Korea between 2016 and 2020 and has been isolated from both dogs and *Haemaphysalis longicornis* ticks, reflecting its long-term circulation and host flexibility [14]. Our findings indicate the presence of reassortant viruses in companion animals, suggesting that reassortment may occur among co-circulating genotypes and potentially contribute to the emergence of genetically diverse strains. Such reassortment may have implications for viral pathogenicity or transmission dynamics in animal hosts [17,18,66], although its biological significance in companion animals remains unclear and requires further investigation. The detection of highly virulent and reassortant SFTS viruses in companion animals raises concerns for individuals in close contact with these animals. Previous sero-epidemiological studies have reported elevated SFTSV seropositivity rates of 2.2-4.2% among veterinarians and veterinary nurses in Japan, even in the absence of clinical symptoms [67,68]. These observations, together with our genomic findings, underscore the need for heightened biosafety awareness in veterinary settings and reinforce the relevance of zoonotic spillover and potential spillback involving animal reservoirs.

Our study identified multiple amino acid substitutions previously reported to be associated with increased fatality in human SFTSV infections. Notably, the Gn-Q341P, Gn-I491M, and RdRp-T1433A substitutions were highly prevalent among the sequenced viruses (97.4%, 74.4%, and 94.9%, respectively), with frequencies markedly higher than those reported in a previous study [34]. These observations may reflect lineage-specific signatures within circulating Korean genotypes or the regional predominance of particular viral lineages. The RdRp-T1433A mutation, which has been associated with increased odds of death in human SFTSV infections (adjusted OR = 2.847), was identified in 37/39 (94.9%) viruses. In addition, the RdRp-R1684K mutation, another variant reported to be associated with increased fatality (adjusted OR = 2.986), was detected in 2/39 (5.1%) viruses (SF24–20 and SF24–85). Interestingly, SF24–72, classified as a genotype D virus, harbored six different amino acid substitutions (one in NS and five in Gn) previously reported to be associated with increased fatality, suggesting that the accumulation of multiple fatality-associated substitutions may not be restricted to traditionally pathogenic genotypes such as B2. However, the potential impact of these mutations on viral fitness or pathogenicity in companion animals requires further investigation. Together, these findings highlight the importance of continued genomic surveillance to monitor genetic variation of SFTSV circulating in companion animals.

We identified seven amino acid mutations within the neutralizing antibody epitope region of the SFTSV Gn-head domain. Notably, the V323I mutation, previously reported as genotype C-specific [36], was also observed in our reassortant virus SF24–19, which carries B2-derived L and S segments and a genotype C-derived M segment. This mutation was also found to be under positive selection in our selection pressure analysis across all SFTSV genotypes, indicating that it may be undergoing adaptive evolution (Table 3). Although residue 323 was identified as part of the B1G11–Gn interface (S5 Table), the V323I substitution did not significantly alter the docking score *in silico*. However, its potential impact on antibody binding affinity *in vivo* requires further experimental validation.

Y83F and G218S mutations were previously associated with genotype B [36]. In our viruses, G218S was present in all genotype B M segments, whereas Y83F was restricted to B2 M segments, suggesting it could serve as a marker for this sub-lineage. Consistent with this, analysis of B2 genotype sequences showed that 152/155 (98.1%) sequences harbored the Y83F substitution, further supporting its potential as a lineage-specific molecular marker. The Y83 residue has also been identified as critical for N1D10 binding in previous alanine substitution analysis, where the Y83A mutant exhibited markedly reduced antibody binding [36]. Based on this, it was expected that the Y83F substitution might similarly have a substantial impact on the antibody binding affinity. However, molecular docking analysis showed that Y83F did not significantly alter antibody binding affinity compared to other mutations within the N1D10 epitope (Fig 4). Structural analysis from the previous study revealed that Y83 is involved in hydrogen bond formation at the interface between the N1D10 antibody and the Gn-head domain [36]. Since phenylalanine (F) lacks the hydroxyl group present in tyrosine (Y) [69], the Y83F substitution may result in the loss of this hydrogen bond. Despite this, the substitution did not appear to substantially impair binding affinity *in silico*, likely because Y83F retains stable π–π interactions with adjacent residues P84 and P122, whereas Y83A disrupts these interactions and may destabilize the loop structure [70]. These compensatory interactions may explain the minimal impact of the Y83F substitution on binding affinity observed in the docking analysis. While the *in* *silico* docking analysis provides preliminary insights into epitope–antibody interactions, these predictions require experimental validation, such as neutralization or binding assays, to confirm their biological relevance.

This study has several limitations. The epidemiological scope of this study was limited by the absence of spatiotemporally matched human- or tick-derived samples, which precluded direct inference of interspecies transmission dynamics within the study regions. Although the most recent human-derived SFTSV genomes from the Republic of Korea in public databases (up to 2023) were included in our analysis, the limited availability of more recent sequences restricts comprehensive comparison with currently circulating strains. Future studies incorporating longitudinal multi-host surveillance and additional human-derived genomes will be necessary to better understand the transmission dynamics and evolutionary trajectories of SFTSV. Such surveillance efforts would benefit from a One Health-based framework that integrates animal and human health data within national surveillance systems. In endemic regions, targeted measures such as routine screening of companion animals, increased awareness among veterinarians, and improved tick control in pets may further strengthen surveillance and prevention efforts. These approaches could facilitate identification of emerging virulent strains, improved tracking of viral evolution, and mitigation of zoonotic risks. In addition, to further validate the clinical significance of our genomic observations, future studies incorporating longitudinal clinical monitoring and comprehensive outcome data are highly warranted. Such prospective approaches will enable a more robust correlation between specific viral mutations and phenotypic disease severity in companion animals.

In conclusion, our study provides novel genomic insights into SFTSV strains circulating in companion animals in the Republic of Korea. The identification of genotype B, including highly virulent and reassortant viruses, together with the detection of multiple amino acid mutations associated with increased fatality, suggests that companion animals may harbor SFTSV strains with enhanced pathogenic potential. The high genetic similarity between animal-derived viruses and those from humans further indicates the likelihood of active viral circulation across species. These findings suggest that companion animals may not only act as incidental hosts but could also contribute to viral maintenance and potential spillover into human populations, highlighting their potential role in SFTSV epidemiology.

## Supporting information

S1 Fig Heatmap of pairwise nucleotide identity (%) for the SFTSV L (A), M (B), and S (C) segments from companion animal cases in the Republic of Korea. Higher identity is shown in more intense red.
(PDF)

S2 Fig Maximum-likelihood phylogenetic trees of the L (A), M (B), and S (C) segments of SFTSV viruses from this study. The scale bar represents the number of substitutions per site. Bootstrap values (≥ 95) are shown at the corresponding nodes. Viruses from this study are highlighted in red. Taxon label colors indicate the country of origin, while the color strip on the right denotes host species. Genotypes are indicated by colored ranges. All trees are midpoint-rooted.
(PDF)

S3 Fig Docking of the S2A5 (A), B1G11 (B), and N1D10 (C) Fabs onto the Gn-head domain of the reference strain WCH/97/HN/China/2011. Antibodies are shown in light blue, and the Gn-head domain is colored light orange. Interface residues are labeled in gray, while key mutated residues identified from our viruses are highlighted in red.
(PDF)

S1 Table Summary of clinical specimens tested for SFTSV infection in the Republic of Korea, April 2023–June 2024. (DOCX)

S2 Table The spectrum of detectable pathogens by Anemia qPCR kit and SFTSV qPCR kit. (DOCX)

S3 Table Primer set used in SFTSV tiling amplicon polymerase chain reaction. (DOCX)

S4 Table Information of clinical samples and genotype analysis obtained in this study. (DOCX)

S5 Table List of interface residues between monoclonal antibodies and the Gn-head domain identified through molecular docking analysis. (DOCX)
