# Supplementary figures and images for: Genomic and phylogenetic characterization of severe fever with thrombocytopenia syndrome virus in companion animals in Korea, 2023–2024
Source: PLoS Negl Trop Dis. 2026 Jun 4;20(6):e0014305. doi: 10.1371/journal.pntd.0014305 (PMC13262934; doi:10.1371/journal.pntd.0014305)

A

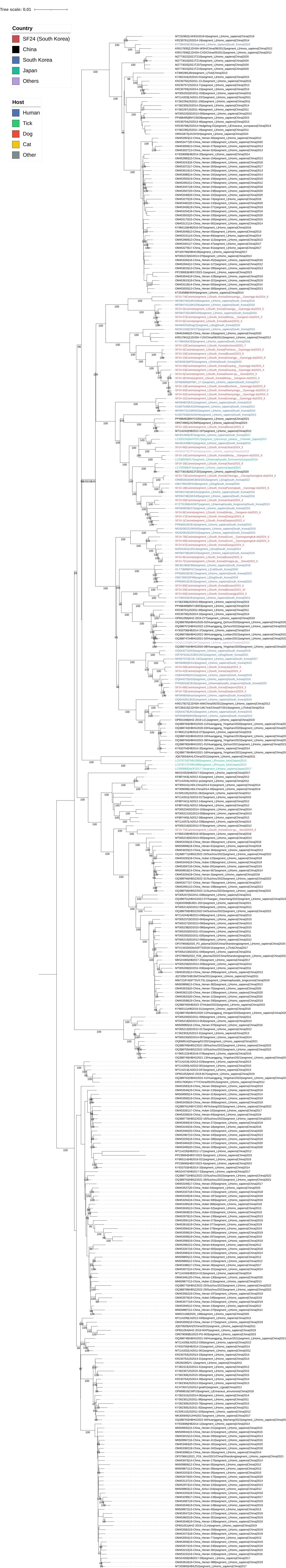

B

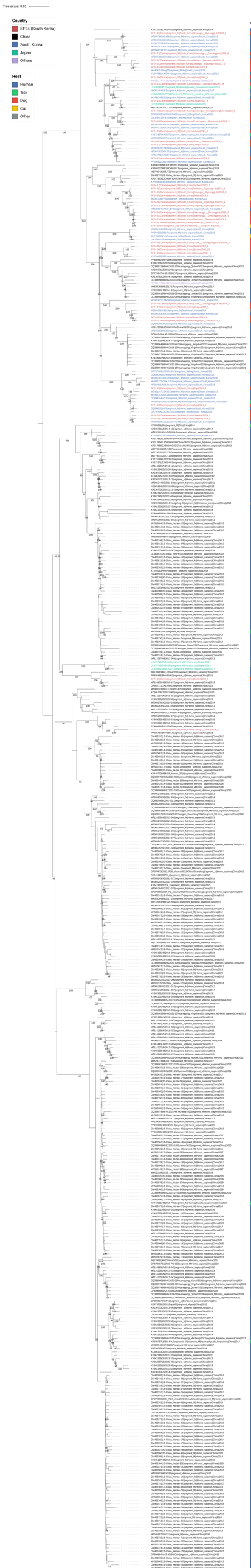

C

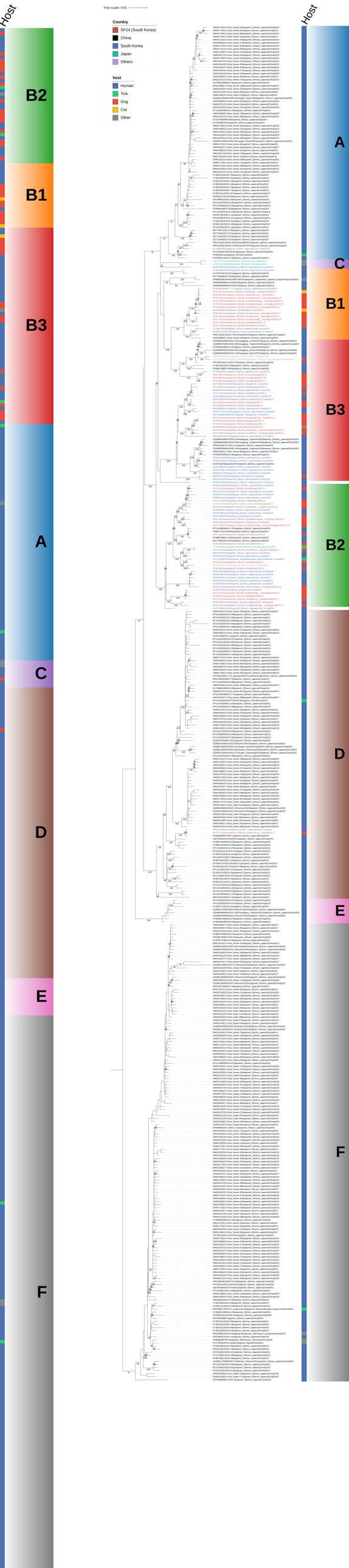

Supplement: S2 Fig — The scale bar represents the number of substitutions per site. Bootstrap values (≥ 95) are shown at the corresponding nodes. Viruses from this study are highlighted in red. Taxon label colors indicate the country of origin, while the color strip on the right denotes host species. Genotypes are indicated by colored ranges. All trees are midpoint-rooted. (PDF) [file pntd.0014305.s002.pdf]
