# Supplementary material for: Genomic and phylogenetic characterization of severe fever with thrombocytopenia syndrome virus in companion animals in Korea, 2023–2024
Source: PLoS Negl Trop Dis. 2026 Jun 4;20(6):e0014305. doi: 10.1371/journal.pntd.0014305 (PMC13262934; doi:10.1371/journal.pntd.0014305)

S3 Fig. Docking of the S2A5 (A), B1G11 (B), and N1D10 (C) Fabs onto the Gn-head domain of the reference strain WCH/97/HN/China/2011. Antibodies are shown in light blue, and the Gn-head domain is colored light orange. Interface residues (≤5 Å) are labeled in gray, and key mutated residues identified from our viruses are highlighted in red.

A

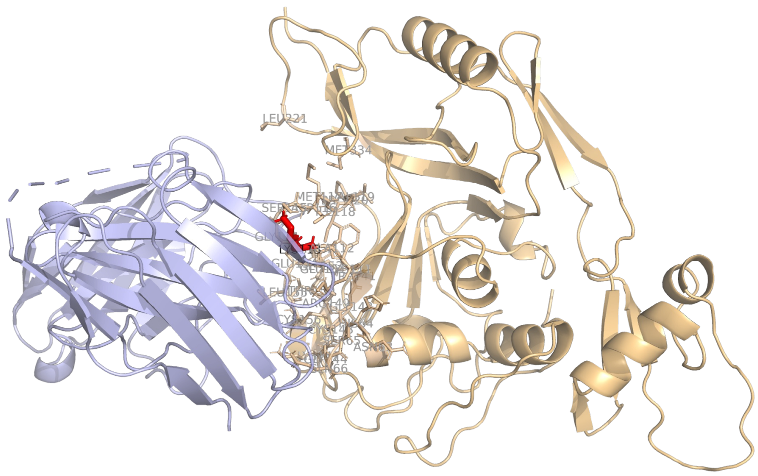

B

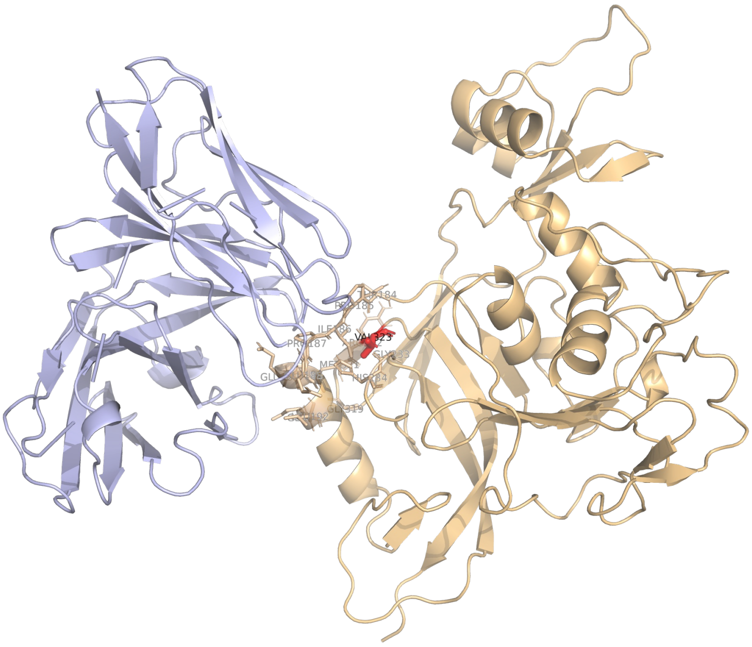

C

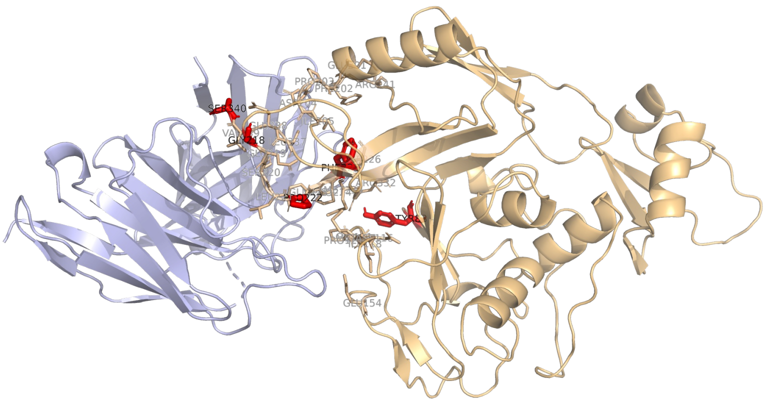

Supplement: S3 Fig — Antibodies are shown in light blue, and the Gn-head domain is colored light orange. Interface residues are labeled in gray, while key mutated residues identified from our viruses are highlighted in red. (PDF) [file pntd.0014305.s003.pdf]
