# Supplementary material for: Genomic and phylogenetic characterization of severe fever with thrombocytopenia syndrome virus in companion animals in Korea, 2023–2024
Source: PLoS Negl Trop Dis. 2026 Jun 4;20(6):e0014305. doi: 10.1371/journal.pntd.0014305 (PMC13262934; doi:10.1371/journal.pntd.0014305)
Supplement: S1 Table — (DOCX) [file pntd.0014305.s004.docx]

S1 Table. Summary of clinical specimens tested for SFTSV infection in the Republic of Korea, April 2023–June 2024.

| Year  Province | 2023^a^ | | | 2024^b^ | | |
| --- | --- | --- | --- | --- | --- | --- |
|  | Tested | Positive | PR^*^ (%) | Tested | Positive | PR (%) |
| Gyeonggi | 583 | 6 | 1.03 | 1,013 | 2 | 0.20 |
| Gangwon | 29 | 3 | 10.34 | 20 | 1 | 5.00 |
| North Chungcheong | 22 | 0 | 0.00 | 49 | 3 | 6.12 |
| South Chungcheong | 40 | 0 | 0.00 | 57 | 0 | 0.00 |
| North Gyeongsang | 34 | 0 | 0.00 | 36 | 0 | 0.00 |
| South Gyeongsang | 32 | 0 | 0.00 | 30 | 2 | 6.67 |
| North Jeolla | 42 | 0 | 0.00 | 34 | 3 | 8.82 |
| South Jeolla | 7 | 0 | 0.00 | 9 | 1 | 11.11 |
| Jeju | 69 | 0 | 0.00 | 30 | 0 | 0.00 |
| Seoul | 307 | 2 | 0.65 | 358 | 14 | 3.91 |
| Incheon | 188 | 1 | 0.53 | 309 | 4 | 1.29 |
| Daejeon | 41 | 1 | 2.44 | 61 | 4 | 6.56 |
| Daegu | 67 | 1 | 1.49 | 42 | 5 | 11.90 |
| Sejong | 6 | 0 | 0.00 | 9 | 2 | 22.22 |
| Ulsan | 19 | 1 | 5.26 | 11 | 0 | 0.00 |
| Busan | 81 | 5 | 6.17 | 86 | 2 | 2.33 |
| Gwangju | 92 | 0 | 0.00 | 43 | 0 | 0.00 |
| Total | 1,659 | 20 | 1.21 | 2,197 | 43 | 1.96 |

PR^*^: Positivity Rate, calculated as ${[The Postitive cases}/{Total tested cases}]\times100$(%)
^a^ The clinical samples were collected from April to December 2023,
^b^ The clinical samples were collected from January to June 2024.
