# Supplementary material for: Genomic and phylogenetic characterization of severe fever with thrombocytopenia syndrome virus in companion animals in Korea, 2023–2024
Source: PLoS Negl Trop Dis. 2026 Jun 4;20(6):e0014305. doi: 10.1371/journal.pntd.0014305 (PMC13262934; doi:10.1371/journal.pntd.0014305)
Supplement: S2 Table — (DOCX) [file pntd.0014305.s005.docx]

S2 Table. The spectrum of detectable pathogens by Anemia qPCR kit and SFTSV qPCR kit.

| Detection kit classification | Kit subtypes | Host | Pathogens | Fluorophore types | Fluorophore threshold |
| --- | --- | --- | --- | --- | --- |
| GCani™ Canine Anemia qPCR Detection Kit | B1 | Dog | *Babesia gibsonii* | FAM | 300 |
|  |  |  | *Leptospira* species | VIC | 300 |
|  |  |  | *Anaplasma* species | Texas Red | 300 |
|  |  |  | *Ehrlichia* species | Cy5.5* | 300 |
|  | B2 | Dog | *Rickettisa* species | FAM | 300 |
|  |  |  | *Bartonella* species | VIC | 300 |
|  |  |  | Hemotropic *Mycoplasma* species | Texas Red | 300 |
|  |  |  | *Borrelia burgdorferi* | Cy5.5 | 300 |
|  | B3 | Dog | *Theileria* species | FAM | 300 |
|  |  |  | *Hepatozoon* species | VIC | 300 |
| GCani™ Feline Anemia qPCR Detection Kit | B1 | Cat | *Babesia felis* | FAM | 300 |
|  |  |  | *Leptospira* species | VIC | 300 |
|  |  |  | *Anaplasma* species | Texas Red | 300 |
|  |  |  | *Ehrlichia* species | Cy5.5 | 300 |
|  | B2 | Cat | *Rickettisa* species | FAM | 300 |
|  |  |  | *Bartonella* species | VIC | 300 |
|  |  |  | Hemotropic *Mycoplasma* species | Texas Red | 300 |
|  |  |  | *Borrelia burgdorferi* | Cy5.5 | 300 |
|  | B3 | Cat | *Theileria* species | FAM | 300 |
|  |  |  | *Hepatozoon* species | VIC | 300 |
|  | V | Cat | Feline leukemia virus | VIC | 300 |
|  |  |  | Feline immunodeficiency virus | FAM | 300 |
|  |  |  | Feline coronavirus | Cy5.5 | 300 |
| GCani™ SFTSV qPCR Detection Kit | - | Universal | Dabe Banda virus (SFTSV) | FAM | 300 |

* Cy5 was used for the fluorophore of internal control for each test to confirm the integrity and success of the amplification process in the experiments performed in this study.
