# Supplementary material for: Genomic and phylogenetic characterization of severe fever with thrombocytopenia syndrome virus in companion animals in Korea, 2023–2024
Source: PLoS Negl Trop Dis. 2026 Jun 4;20(6):e0014305. doi: 10.1371/journal.pntd.0014305 (PMC13262934; doi:10.1371/journal.pntd.0014305)
Supplement: S3 Table — (DOCX) [file pntd.0014305.s006.docx]

S3 Table. Primer set used in SFTSV tiling amplicon polymerase chain reaction.

| Segment | Name | Sequence | Length | %GC | Tm | Start | Stop | Amplicon Length |
| --- | --- | --- | --- | --- | --- | --- | --- | --- |
| L | L-1-L | ACACAGAGACGCCCAGATGA | 20 | 55 | 60.9 | -15 | 4 | 624 |
|  | L-1-R | GTGTAGATCTCATTGGCTATGCAGA | 25 | 44 | 60.5 | 584 | 608 |  |
|  | L-2-L | GTGTTGATATCATGGAGAACCCGA | 24 | 45.8 | 60.4 | 467 | 490 | 625 |
|  | L-2-R | AGCTCACCATATGCGCCATG | 20 | 55 | 60.9 | 1072 | 1091 |  |
|  | L-3-L | GGCTAGAGATCAATAGATGTGAGGC | 25 | 48 | 60.6 | 887 | 911 | 601 |
|  | L-3-R | GTGTCTTGCATGGCAGATGC | 20 | 55 | 60.2 | 1468 | 1487 |  |
|  | L-4-L | AGAAGTGGAAGAGAATGGCAGC | 22 | 50 | 60.6 | 1292 | 1313 | 591 |
|  | L-4-R | CCAGCTTCTCTCTCCAGAAAGC | 22 | 54.5 | 60.7 | 1861 | 1882 |  |
|  | L-5-L | GGGAATGGGAGCTAACAGAGTTC | 23 | 52.2 | 60.7 | 1772 | 1794 | 624 |
|  | L-5-R | GCTTCTTAGGGGAATCAGTGTCC | 23 | 52.2 | 60.7 | 2373 | 2395 |  |
|  | L-6-L | GGGTACTACAAGAACAAAGAGGAGTC | 26 | 46.2 | 60.8 | 2257 | 2282 | 601 |
|  | L-6-R | CAACAGTCTCATGTGGGCTCA | 21 | 52.4 | 60.3 | 2837 | 2857 |  |
|  | L-7-L | AAAAGAATCAGCATGGTGGCCT | 22 | 45.5 | 60.8 | 2735 | 2756 | 599 |
|  | L-7-R | CATGTAGCCCTCCTTGAGCTTC | 22 | 54.5 | 60.7 | 3312 | 3333 |  |
|  | L-8-L | AGACAGAGACAGGGATGATGCA | 22 | 50 | 60.9 | 3218 | 3239 | 592 |
|  | L-8-R | TCTGGGTCTGATATCAGCATCCC | 23 | 52.2 | 61.3 | 3787 | 3809 |  |
|  | L-9-L | TCAGAGAAATCAACAGTGAACACAGT | 26 | 38.5 | 60.9 | 3502 | 3527 | 599 |
|  | L-9-R | TCATCTATCTGCTCCACCCAGT | 22 | 50 | 60.4 | 4079 | 4100 |  |
|  | L-10-L | CTCAGCCACTCTGTTATGGTGT | 22 | 50 | 60 | 4000 | 4021 | 584 |
|  | L-10-R | TTAGTCCTTTTCAGGCCAAACCA | 23 | 43.5 | 60.4 | 4561 | 4583 |  |
|  | L-11-L | AGAGAAACATTGTCAGGAGCCG | 22 | 50 | 60.6 | 4475 | 4496 | 613 |
|  | L-11-R | TGTGCCCTCTCAATCTGGTCTA | 22 | 50 | 60.6 | 5066 | 5087 |  |
|  | L-12-L | CTCTTGTGATACCTCAGCCATCAG | 24 | 50 | 60.7 | 4967 | 4990 | 604 |
|  | L-12-R | ATATCAGTGTCTCTCGGCCTGT | 22 | 50 | 60.7 | 5549 | 5570 |  |
|  | L-13-L | ACCCAGATGAGGTCAAGATGAGA | 23 | 47.8 | 60.6 | 5459 | 5481 | 611 |
|  | L-13-R | ACACATTGTGATGGCAGCTTG | 21 | 47.6 | 59.7 | 6049 | 6069 |  |
|  | L-14-L | CAGCTGGATCTGCTTGAAAACTC | 23 | 47.8 | 60.1 | 5643 | 5665 | 709 |
|  | L-14-R | CACAAAGACCGCCCAGATCT | 20 | 55 | 60 | 6332 | 6351 |  |
| M | M-1-L | ACACAGAGACGGCCAACA | 18 | 55.6 | 58.8 | -17 | 0 | 560 |
|  | M-1-R | TGGCAAGCAACATCACCTATCC | 22 | 50 | 60.9 | 521 | 542 |  |
|  | M-2-L | TTGGGATGCTTGTCGTGAAGAA | 22 | 45.5 | 60.5 | 311 | 332 | 502 |
|  | M-2-R | TCTTCTGATTCAGAACACGGCC | 22 | 50 | 60.6 | 791 | 812 |  |
|  | M-3-L | TTGTGTGCTACAAGGAAGGGAC | 22 | 50 | 60.5 | 767 | 788 | 507 |
|  | M-3-R | TGCAACTGCCCTCAAATGTGA | 21 | 47.6 | 60.8 | 1253 | 1273 |  |
|  | M-4-L | CAAGTGGCAAGAAAAGCACGG | 21 | 52.4 | 60.9 | 1154 | 1174 | 504 |
|  | M-4-R | TCAGGATGACAGGTGAGTACATC | 23 | 47.8 | 59.3 | 1635 | 1657 |  |
|  | M-5-L | TAGGGGAAGGCAAGTGATCCAT | 22 | 50 | 60.9 | 1533 | 1554 | 505 |
|  | M-5-R | CCTGTCCATTTTACCTGCCCAA | 22 | 50 | 60.8 | 2016 | 2037 |  |
|  | M-6-L | ACCCAGGACAAGAGGCATGT | 20 | 55 | 61.1 | 1793 | 1812 | 493 |
|  | M-6-R | ACACTCACACCCTTGAAGACTTG | 23 | 47.8 | 60.7 | 2263 | 2285 |  |
|  | M-7-L | CAGTCATAGAGCTAACAATGCCCT | 24 | 45.8 | 60.4 | 2195 | 2218 | 507 |
|  | M-7-R | GGCTCAGTCGAAGGCCTCTATA | 22 | 54.5 | 61.1 | 2680 | 2701 |  |
|  | M-8-L | AGGTTTGAGAGAAGCCATGACA | 22 | 45.5 | 59.6 | 2593 | 2614 | 666 |
|  | M-8-R | GCTCCCAGAGGGTCACTAGA | 20 | 60 | 60 | 3239 | 3258 |  |
| S | S-1-L | ACCCCCTTCATTTGGAAACC | 20 | 50 | 58 | -19 | 0 | 538 |
|  | S-1-R | AGGATCTCAGGTAACCCAAGTCT | 23 | 47.8 | 60.2 | 496 | 518 |  |
|  | S-2-L | GAACAGCTGGGCAATGGAAAC | 21 | 52.4 | 60.3 | 363 | 383 | 471 |
|  | S-2-R | ATGGTGTTTAGGGCTGCAGTTC | 22 | 50 | 61.1 | 812 | 833 |  |
|  | S-3-L | TTCACTTCTTCCTCATTGCGTAAG | 24 | 41.7 | 59.3 | 610 | 633 | 460 |
|  | S-3-R | TGCTGCTGTGAACTCTGTCTTC | 22 | 50 | 60.8 | 1048 | 1069 |  |
|  | S-4-L | TAAGCAGCAGCAGCAACCTC | 20 | 55 | 61 | 950 | 969 | 458 |
|  | S-4-R | TATGGACTGGTTGAGAGGGCAG | 22 | 54.5 | 61.5 | 1386 | 1407 |  |
|  | S-5-L | GTTGGAATCAGGGACCCAAAGG | 22 | 54.5 | 61.1 | 1211 | 1232 | 487 |
|  | S-5-R | AGGAAAGACGCAAAGGAGTGA | 21 | 47.6 | 59.6 | 1677 | 1697 |  |
