# Supplementary material for: Genomic and phylogenetic characterization of severe fever with thrombocytopenia syndrome virus in companion animals in Korea, 2023–2024
Source: PLoS Negl Trop Dis. 2026 Jun 4;20(6):e0014305. doi: 10.1371/journal.pntd.0014305 (PMC13262934; doi:10.1371/journal.pntd.0014305)
Supplement: S4 Table — (DOCX) [file pntd.0014305.s007.docx]

S4 Table. Information of clinical samples and genotype analysis obtained in this study.

| Strain | Host | Source | Country | Location | Date | Ct Value | L | M | S | Segment Types | Genotype |
| --- | --- | --- | --- | --- | --- | --- | --- | --- | --- | --- | --- |
| SF24-1 | canine | serum | South Korea | Daejeon | 2023.4 | 21.58 | B2 | B2 | B2 | B2/B2/B2 | B2 |
| SF24-2 | canine | serum | South Korea | Gwangju, Gyeonggi-do | 2023.5 | 26.43 | B1 | B2 | B2 | B1/B2/B2 | Reassortant (R11) |
| SF24-3 | feline | serum | South Korea | Wonju, Gangwon-do | 2023.4 | 19.72 | B1 | B1 | B1 | B1/B1/B1 | B1 |
| SF24-4 | canine | serum | South Korea | Pocheon, Gyeonggi-do | 2023.5 | 22.6 | B1 | B1 | B1 | B1/B1/B1 | B1 |
| SF24-5 | canine | serum | South Korea | Nowon-gu, Seoul | 2023.5 | 25.02 | B1 | B1 | B1 | B1/B1/B1 | B1 |
| SF24-6 | canine | serum | South Korea | Busan | 2023.6 | 20.48 | B1 | B2 | B2 | B1/B2/B2 | Reassortant (R11) |
| SF24-7 | canine | serum | South Korea | Songpa-gu, Seoul | 2023.5 | 35.11 | B3 | B3 | B3 | B3/B3/B3 | B3 |
| SF24-8 | canine | serum | South Korea | Wonju, Gangwon-do | 2023.6 | 19.73 | B2 | B2 | B2 | B2/B2/B2 | B2 |
| SF24-9 | canine | serum | South Korea | Busan | 2023.6 | 28.78 | B3 | B3 | B3 | B3/B3/B3 | B3 |
| SF24-10 | canine | serum | South Korea | Busan | 2023.6 | 19.24 | B1 | B1 | B1 | B1/B1/B1 | B1 |
| SF24-11 | canine | serum | South Korea | Gwangju, Gyeonggi-do | 2023.6 | 28.03 | B2 | B2 | B2 | B2/B2/B2 | B2 |
| SF24-12 | canine | serum | South Korea | Incheon | 2023.7 | 24.89 | B1 | B1 | B1 | B1/B1/B1 | B1 |
| SF24-13 | canine | serum | South Korea | Bucheon, Gyeonggi-do | 2023.8 | 26.35 | B1 | B1 | B1 | B1/B1/B1 | B1 |
| SF24-14 | canine | serum | South Korea | Wonju, Gangwon-do | 2023.8 | 23.05 | B2 | B2 | B2 | B2/B2/B2 | B2 |
| SF24-15 | canine | serum | South Korea | Gwangju, Gyeonggi-do | 2023.9 | 28.15 | B1 | B1 | B1 | B1/B1/B1 | B1 |
| SF24-16 | canine | serum | South Korea | Ulsan | 2023.9 | 19.46 | B2 | B2 | B2 | B2/B2/B2 | B2 |
| SF24-17 | canine | serum | South Korea | Daegu | 2023.9 | 25.24 | B2 | B2 | B2 | B2/B2/B2 | B2 |
| SF24-18 | canine | serum | South Korea | Pyeongtaek, Gyeonggi-do | 2023.9 | 32.78 | B2 | B2 | B2 | B2/B2/B2 | B2 |
| SF24-19 | canine | serum | South Korea | Busan | 2023.9 | 30.32 | B2 | C | B2 | B2/C/B2 | Reassortant (R10) |
| SF24-20 | canine | serum | South Korea | Busan | 2023.1 | 25.88 | B3 | B3 | B3 | B3/B3/B3 | B3 |
| SF24-42 | canine | serum | South Korea | Jeju | 2024.4 | 20.85 | B3 | B3 | B3 | B3/B3/B3 | B3 |
| SF24-53 | canine | serum | South Korea | Goyang, Gyeonggi-do | 2024.4 | 27.6 | B1 | B1 | B1 | B1/B1/B1 | B1 |
| SF24-55 | canine | serum | South Korea | Ulsan | 2024.4 | 30.91 | B2 | B2 | B2 | B2/B2/B2 | B2 |
| SF24-56 | canine | serum | South Korea | Goyang, Gyeonggi-do | 2024.4 | 27.36 | B1 | B1 | B1 | B1/B1/B1 | B1 |
| SF24-57 | canine | serum | South Korea | Wonju, Gangwon-do | 2024.5 | 27.02 | B1 | B2 | B2 | B1/B2/B2 | Reassortant (R11) |
| SF24-59 | canine | serum | South Korea | Jeju | 2024.5 | 24.06 | B3 | B3 | B3 | B3/B3/B3 | B3 |
| SF24-60 | canine | serum | South Korea | Gwangju | 2024.5 | 27.9 | B3 | B3 | B3 | B3/B3/B3 | B3 |
| SF24-62 | canine | serum | South Korea | Namyangju, Gyeonggi-do | 2024.5 | 19.17 | B1 | B1 | B1 | B1/B1/B1 | B1 |
| SF24-64 | canine | serum | South Korea | Namyangju, Gyeonggi-do | 2024.5 | 27.71 | B1 | B1 | B1 | B1/B1/B1 | B1 |
| SF24-66 | canine | serum | South Korea | Ulsan | 2024.5 | 18.47 | B2 | B2 | B2 | B2/B2/B2 | B2 |
| SF24-67 | canine | serum | South Korea | Daegu | 2024.5 | 19.79 | B3 | B3 | B3 | B3/B3/B3 | B3 |
| SF24-68 | canine | serum | South Korea | Daejeon | 2024.5 | 19.08 | B3 | B3 | B3 | B3/B3/B3 | B3 |
| SF24-69 | canine | serum | South Korea | Gumi, Gyeongsangbuk-do | 2024.5 | 23.22 | B3 | B3 | B3 | B3/B3/B3 | B3 |
| SF24-70 | canine | serum | South Korea | Daejeon | 2024.5 | 18.17 | B3 | B3 | B3 | B3/B3/B3 | B3 |
| SF24-72 | canine | serum | South Korea | Guro-gu, Seoul | 2024.6 | 20.66 | D | D | D | D/D/D | D |
| SF24-73 | canine | serum | South Korea | Cheongju, Chungcheongbuk-do | 2024.6 | 21.55 | B2 | B2 | B2 | B2/B2/B2 | B2 |
| SF24-74 | canine | serum | South Korea | Namyangju, Gyeonggi-do | 2024.6 | 22.16 | B1 | B2 | B2 | B1/B2/B2 | Reassortant (R11) |
| SF24-78 | canine | serum | South Korea | Gumi, Gyeongsangbuk-do | 2024.6 | 17.98 | B3 | B3 | B3 | B3/B3/B3 | B3 |
| SF24-85 | canine | serum | South Korea | Busan | 2024.6 | 25.52 | B3 | B3 | B3 | B3/B3/B3 | B3 |
