# Supplementary material for: Genomic and phylogenetic characterization of severe fever with thrombocytopenia syndrome virus in companion animals in Korea, 2023–2024
Source: PLoS Negl Trop Dis. 2026 Jun 4;20(6):e0014305. doi: 10.1371/journal.pntd.0014305 (PMC13262934; doi:10.1371/journal.pntd.0014305)
Supplement: S5 Table — (DOCX) [file pntd.0014305.s008.docx]

S5 Table. List of interface residues (≤5 Å) between monoclonal antibodies and the Gn-head domain identified through molecular docking analysis.

| Antibody | Interface residues* |
| --- | --- |
| S2A5 | ASN63, HIS64, SER65, GLN66, LYS111, ALA112, **LYS113****, GLY114, SER115, ASP116, MET117, ILE118, VAL119, PRO120, TRP141, GLY142, CYS143, GLY144, LYS147, ARG149, THR150, GLU151, SER152, GLY153, GLU154, LEU155, CYS156, LEU221, MET334 |
| B1G11 | THR184, PRO185, ILE186, PRO187, GLU188, GLU189, THR190, LEU192, GLU193, GLY233, HIS234, GLY319, MET321, ARG322, **VAL323** |
| N1D10 | **TYR83**, ASP116, MET117, ILE118, PRO120, GLY121, GLU154, GLU201, PHE202, PRO203, ASP204, ILE205, **GLY218**, GLU219, SER220, LEU221, **PRO222**, GLN223, PRO224, **PHE225**, ASP226, ARG241, ARG332, MET334, LEU337, GLU338, VAL339, **SER340** |

* Interface residues were defined as residues within 5 Å of any atom of the docking partner in the final complex structures.

** Key amino acid mutations within antibody-targeted epitope regions identified by epitope sequence variation analysis are indicated in bold.
